# Varietal Dynamics and Yam Agro-Diversity Demonstrate Complex Trajectories Intersecting Farmers’ Strategies, Networks, and Disease Experience

**DOI:** 10.3389/fpls.2016.01962

**Published:** 2016-12-23

**Authors:** Laurent Penet, Denis Cornet, Jean-Marc Blazy, Angela Alleyne, Emilie Barthe, François Bussière, Sébastien Guyader, Claudie Pavis, Dalila Pétro

**Affiliations:** ^1^INRA, UR1321, ASTRO Agrosystèmes TropicauxGuadeloupe, France; ^2^CIRAD, UMR AGAPGuadeloupe, France; ^3^Department of Biological and Chemical Sciences, Cave Hill Campus – University of the West IndiesBridgetown, Barbados

**Keywords:** agro-diversity dynamics, anthracnose, *Colletotrichum gloeosporioides*, cultivar turnover, *Dioscorea*, landraces, varieties, yams

## Abstract

Loss of varietal diversity is a worldwide challenge to crop species at risk for genetic erosion, while the loss of biological resources may hinder future breeding objectives. Loss of varieties has been mostly investigated in traditional agricultural systems where variety numbers are dramatically high, or for most economically important crop species for which comparison between pre-intensive and modern agriculture was possible. Varietal dynamics, i.e., turnover, or gains and losses of varieties by farmers, is nevertheless more rarely studied and while we currently have good estimates of genetic or varietal diversity for most crop species, we have less information as to how on farm agro-diversity changes and what cause its dynamics. We therefore investigated varietal dynamics in the agricultural yam system in the Caribbean island of Guadeloupe. We interviewed producers about varieties they cultivated in the past compared to their current varieties, in addition to characterizing yam cropping characteristics and both farm level and producers socio-economic features. We then used regression tree analyses to investigate the components of yam agro-diversity, varietal dynamics and impact of anthracnose on varieties. Our data demonstrated that no dramatic loss of varieties occurred within the last decades. Cultivation changes mostly affected widespread cultivars while frequency of uncommon varieties stayed relatively stable. Varietal dynamics nevertheless followed sub-regional patterns, and socio-economic influences such as producer age or farm crop diversity. Recurrent anthracnose epidemics since the 1970s did not alter varietal dynamics strongly, but sometimes translated into transition from *Dioscorea alata* to less susceptible species or into a decrease of yam cultivation. Factors affecting changes in agro-diversity were not relating to agronomy in our study, and surprisingly there were different processes delineating short term from long term varietal dynamics, independently of disease risk. Our results highlighted the importance of understanding varietal dynamics, an often overlooked component of agriculture sustainability, in addition to evolutionary forces shaping agro-diversity and genetic diversity distribution within crops. It is also crucial to understand how processes involved do scale up worldwide and for different crop species, so as not to mislead on-farm conservation efforts and efficacy of agro-diversity preservation.

## Introduction

Modern intensification of agriculture has driven a standardization of varieties toward elite cultivars with greater productivity (Brown, 2010; van de Wouw et al., 2010). This transition resulted in a reduction of cultivated varieties in most crop species impacted by agriculture transition toward higher and more homogenous productivity. Many studies have investigated the loss of varieties in diverse crop systems (Berg, 2009), confirming a global pattern of loss of traditional varieties (e.g., Martos et al., 2005; Willemen et al., 2007), while supporting the active role of farmers in shaping and maintaining crop diversity, both at species (Brown, 2010) and variety levels (Chaïr et al., 2010). Genetic erosion is defined as the loss of diversity resulting from reducing variety stock as a result of transitioning toward agricultural modernization. It is the main threat to cultivar breeding programs which are dependent on diversity in pools of potential variety progenitors (e.g., Golding and Falk, 2010). Genetic erosion occurs along with loss of landraces (Hammer et al., 1996; Zeven, 1998; Tsegaye and Berg, 2007), though some studies have mitigated concerns about the extent of the genetic loss threat (e.g., Bellon, 1996; Khlestkina et al., 2004; Huang et al., 2007), suggesting that diversity and variety loss dynamics may stabilize after the transition toward more intensive agriculture has occurred (van de Wouw et al., 2010).

While agronomic concerns about genetic resources have been met with a growing interest as a research focus (e.g., Tsegaye and Berg, 2007) and is accompanied by elaborate protocols of landraces conservation in gene banks by specialized agronomic institutes around the world, more recently it also elicited research on pathways to *in situ* resources management (on farm conservation efforts, Brush, 1991; Maxted et al., 2002; Seboka and van Hintum, 2006; Thomas et al., 2012). Indeed, there are numerous ways by which farmers impact the nature and structure of cultivated varieties, including their own preferences for productivity or stability of production (Asrat et al., 2010), taste for a diverse array of cultivars or a more focused mono-varietal cropping system (Perales et al., 2003; Montero-Rojas et al., 2011), seed provisioning –whether via seed producers, traditional or occasional exchanges with other producers (Elias et al., 2001), locally or at greater scales (Zimmerer and Douches, 1991) and whenever they self-select their own seeds in the fields (Dansi et al., 1999; Dansi et al., 2013). As a result, crops and variety diversity are often correlated to geographical patterns (e.g., Enjalbert et al., 2011; Thomas et al., 2012) or cultural practices (Claid, 2011), with sometimes complex interaction modalities (Zannou et al., 2009), thus contrasting with trends of homogeneity constraints usually associated with modern production standards (Zeven, 1998).

Many studies have characterized impact of farmer variety management at the genetic level (e.g., Busso et al., 2000; Vargas-Ponce et al., 2009), emphasizing the importance of gene flow, especially in traditional allogamous crops (e.g., Barnaud et al., 2006; Claid, 2011) and between cultivated landraces and wild relatives of crops (Jarvis and Hodgkin, 1999; Barnaud et al., 2009). Indeed, sexual reproduction cycles are fundamental to reshuﬄing coexisting diversity, therefore creating new combinations of traits and potential new varieties (Scarcelli et al., 2006), or increase diversity within existing varieties when seedlings are incorporated in already existing varieties (Elias et al., 2001). This process is known to occur in clonal crops as well (e.g., Chaïr et al., 2010; Scarcelli et al., 2013), and is probably more easily managed because of clonality (Scarcelli et al., 2011), notwithstanding other process leading to the multi-genotypic nature of clonal varieties in most traditional clonal crops involving seeds exchanges and blurring phenotypic boundaries (Zimmerer and Douches, 1991). Although clonal crops are known to be generally diverse, their diversity is strongly impacted by producer communities (e.g., Baco et al., 2008) and it demonstrates a higher susceptibility to genetic turnover than their allogamous counterparts, making conservation policies more complex to implement (Moscoe et al., 2016).

In contrast with the numerous studies dealing with genetic diversity and farmer management impacts on genetic resources, far less work has been conducted on landraces and varietal dynamics (Berg, 2009). While varieties are correctly interpreted as transient collections of genotypes more or less unified under a phenotypic template and subjected to dynamic genetic evolution over time (Busso et al., 2000), much less is known about temporal dynamics of varieties, especially from early genesis to their eventual loss. Variety adoption by farmers demonstrably correlates with agronomic characteristics, usually pertaining to productivity (Bellon, 1996), resistance to pests or adverse cropping conditions (Mulumba et al., 2012; Sajise et al., 2012), environmental conditions (Bamire and Amujoyegbe, 2005) but also to stability of production (Altieri, 2009). Elite modern cultivars are usually thought to combine these traits, yet producers usually lean toward averaging risks and manage varieties that match up with expected performances (e.g., Duputié et al., 2009). While adoption of new varieties is a factor in global variety dynamics (Sperling and Loevinsohn, 1993), producers’ choice to maintain or abandon varieties (Wood and Lenne, 1997; Teklu and Hammer, 2006) comes as a balance that also critically needs to be addressed. Indeed, little is known about variety turnover (but see Brennan and Byerlee, 1991; Sperling and Loevinsohn, 1993). Gains and losses are nevertheless key components of varietal dynamics and agrobiodiversity, and need special focus because this issue is still very poorly addressed.

We therefore investigated this issue with Water Yam crop (*Dioscorea alata* L.) in Guadeloupe, and aimed to describe gains and losses in cultivated varieties over the previous decades. In modern and intensive agricultural settings, disease is the main driver of varietal turnover, since evolving pathogens eventually adapt to varieties and impose renewal of previously resistant cultivars. Yam main disease in the Caribbean, anthracnose, is cause by the fungus *Colletotrichum gloeosporioides* and is eventually leading to extensive defoliation and dramatic yield losses in the fields (Winch et al., 1984). We thus also explored the impact of anthracnose disease on varietal dynamics in this crop species complex. We did not address genetic erosion within varieties, but focused directly on variety gains and losses by producers. More specifically, we sought to address the following questions: Did yam agro-diversity decline locally over the three most recent decades? What factors structured past and current agro-diversity, and how did they affect loss and gain dynamics? Did recurrent epidemics of anthracnose disease since the 1970s actually impact agro-diversity dynamics and how?

## Materials and Methods

**FIGURE 1:**
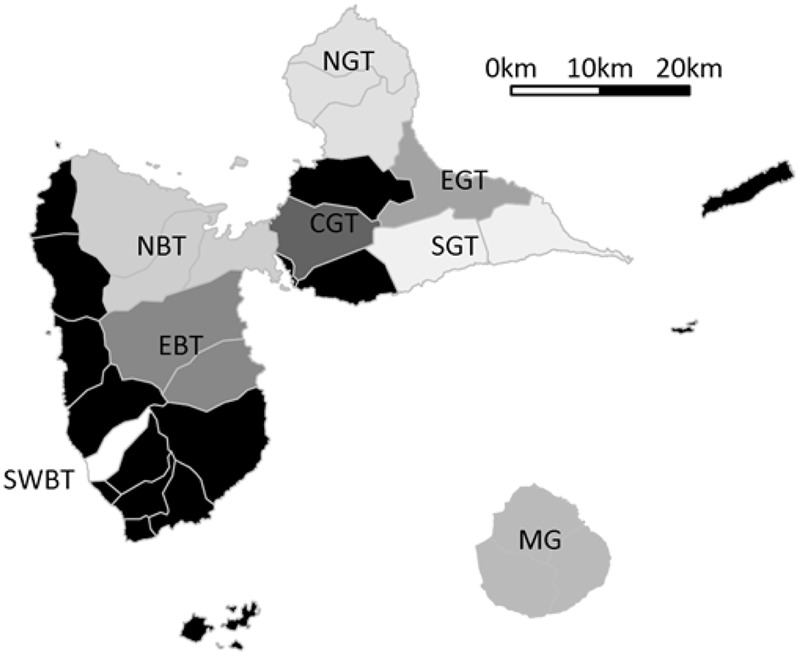
**Sub-regions prospected for yam agro-diversity.** Each area is named after its location: South West Basse Terre (SWBT), East Basse Terre (EBT), North Basse Terre (NBT), the island of Marie Galante (MG), South Grande Terre (SGT), center of Grande Terre (CGT), East Grande Terre (EGT), and North Grande Terre (NGT). Non prospected areas are indicated in black.

In 2014, we conducted interviews with a sample of 78 yam producers aimed at investigating their perception of anthracnose (Penet et al., 2016). Interviews were conducted with volunteer producers specifically agreeing for the interview, usually officially registered as yam growers by the Agriculture chamber, usually with prior commitment to participating in agronomic research and agricultural censuses, with full disclosure and educated informed prior consent with regard to the aims of the research project and use of resulting data, with guaranteed anonymization, respected confidentiality when requested and freedom to retract any time and without justification. During these interviews, we asked questions about the diversity of *D. alata* varieties and other yam species that they were growing. We specifically asked them about their current varieties or species, and also the varieties they used to grow in the past and the previous year (past cultivation spanning their respective careers, i.e., three decades on average), allowing us to contrast variety losses and gains. The dependent variables in our analyses were thus number of varieties cultivated in the past, number of currently cultivated varieties, long term varietal diversity dynamics (current minus past), short term varietal diversity dynamics (current minus previous year), total number of varieties for *D. alata*, and number of species other than *D. alata*. Alongside varietal and species diversity, we recorded cropping system and farm characteristics. Geography was divided into sub-regions for both pedo-climatic parts of Guadeloupe (‘Basse Terre’, a volcanic and humid area, and ‘Grande Terre’, a dry and calcareous area), subsequently divided by relevant cardinal point for splitting convenience (**Figure 1**): from South West Basse Terre (SWBT), East Basse Terre (EBT), North Basse Terre (NBT), the island of Marie Galante (MG), South Grande Terre (SGT), Centre Grande Terre (CGT), East Grande Terre (EGT), and North Grande Terre (NGT). Areas with only casual yam cultivation were not prospected (North West coast of Basse Terre, “Banana Belt” Southern Basse Terre, and dependencies of La Désirade and Les Saintes). We also collected information on farm and cropping characteristics (**Table 1**).

**Table 1:** Covariates used in regression trees for past and current varietal diversity and varietal dynamics.

Covariate	Nature	Comment	Expected impact on agro-diversity (see Penet et al., 2016 for fine analysis)
Number of varieties cultivated in the past	Quantitative		Dependent variables, except for analyzing impact of anthracnose disease
Number of currently cultivated varieties	Quantitative		
Long term varietal diversity dynamics	Quantitative	Calculated as (current minus past)	
Short term varietal diversity dynamics	Quantitative	Calculated as (current minus previous year)	
Number of other species cultivated in the past	Quantitative		
Number of currently cultivated other species	Quantitative		
Long term species diversity dynamics	Quantitative	Calculated as (current minus past)	
Short term species diversity dynamics	Quantitative	Calculated as (current minus previous year)	
Age of producer	Quantitative		No prediction (experience and access to diversity increase with age, and possibly decrease at end of career)
Cultures (number of crops)	Quantitative		Diversity begets yam diversity (polyculture vs. monoculture)
Epidemics (experience of anthracnose disease)	Quantitative	Number of past epidemics that dramatically reduced yam harvest	Expected to decrease diversity due to loss of sensible varieties
Financial satisfaction with yam crop	Binary (0 = no, 1 = yes)	Satisfaction with crop monetary returns	No prior prediction
Frequency of chemicals use	Semi quantitative	Index scaled on a monthly basis	Tend to be associated with intensive cropping, therefore correlated to a decrease in diversity
Intensity of weeding	Semi quantitative	Index scaled from none to mechanical to chemical weeding	No prior prediction
Number of field plots devoted to yam crop	Quantitative		Correlated to yam diversity (opportunities for more varieties)
Personal satisfaction with yam crop	Binary		Correlated to yam diversity
Previous crop	Alphanumeric	Bananas, fallow, gardening, grazing, sugarcane, tubers, yam or not answered (na)	No prior prediction
Readiness to invest financially in yam crop	Binary	producer can afford costs due to unexpected events	No prior prediction
SAU (total surface cultivated)	Quantitative	Proxy for farm size	Tend to be associated with intensive cropping, therefore correlated to a decrease in diversity
Seed tuber selection criteria for yam	Binary	Choice made at plantation (estimate of tuber quality)	No prior prediction, but possibly associated with greater care, so indirectly associated with diversity via buffering varietal loss
Seed tuber size criteria for yam	Binary	Choice made at plantation	
Seed tuber treatment before planting	Binary	Preventive disease management strategy	
Staking	Binary		No prior prediction, but correlated with cultivation of other species
Sub-region	Alphanumeric	South West Basse Terre (SWBT), East Basse Terre (EBT), North Basse Terre (NBT), island of Marie Galante (MG), South Grande Terre (SGT), Centre Grande Terre (CGT), East Grande Terre (EGT), North Grande Terre (NGT) (see **Figure 1**)	Basse Terre location as a center of diversity and traditional yam cropping, NBT as ‘Yam Belt’
Use of chemicals	Binary	Disease and weed management relies on chemicals	No prior prediction, but possibly associated with greater care, so indirectly associated with diversity via buffering varietal loss
Use of fertilizer	Binary		
Workload	Semi quantitative	Reported yam crop workload, semi quantitative index based on effort during cropping season	
Yam dynamics (commitment to yam production)	Semi quantitative	Index scaled for future commitment, from decrease, undecided, stable to increase in future cultivated surface	Decreased commitment expected to correlate with lower varietal diversity or greater loss of varieties

### Varietal Diversity: Past and Current State for Yams in Guadeloupe

We examined variety reports with a list of 20 varieties of *D. alata* and other species (*D. rotundata-cayenensis* complex, *D. trifida*, and *D. esculenta*) cultivated in Guadeloupe. We then ranked them from most frequent to rare based on past cultivation (excluding variation from previous year), and contrasted past cultivation frequencies to current cultivation frequencies. We further assessed variety censuses from previous unpublished studies from 2005 and 2010, to investigate the impact of sample size on our frequency estimates and assessed for sampling bias in our data set (Defèche, Voisin, personal communications, *N* = 69 and 37, respectively). We tested for variety and other yams species differences between current and past variety numbers using ‘Welch unequal variances *t*-test’ with R software (R Core Team, 2012) in order to account for greater dispersion around means with regard to varieties cultivated in the past. Welch *t*-test is usually used when both sample size and variance are unequal (our sample size was balanced), but the procedure is demonstrably robust and without loss of statistical power when equality of variances and sample size conditions required for a regular *t*-test are met (Ruxton, 2006; Fagerland, 2012).

### Varietal Dynamics in Guadeloupe

We then used cropping system characteristics to explain variety and species diversity with emphasis on past and current yam cultivation. We used a regression tree approach to find the covariates that best explained past agro-diversity, current agro-diversity and trends in varietal gains and loss. We conducted the analyses for water yam and for other yam species separately.

Classification and regression trees are a family of machine learning statistical techniques ideally suited both for exploring and analyzing relationships between factors when nonlinear or high-order interactions may characterize data structure (Breiman et al., 1984). Regression tree methods are non-parametric regression approaches consisting in recursive partitioning of data based on the most homogenous resulting group splits (De’Ath, 2002; Strobl et al., 2009). This method has numerous advantages: it is not affected by variance differentials compared to classical parametric analyses; it is generally not strongly affected by outliers; without the need to transform or edit the dataset beyond fixing typographs; it can deal with missing data and accommodates mixed data (for example binary, categorical, quantitative) and high dimension datasets. Last, exploratory tree models are graphically easy to interpret (De’ath and Fabricius, 2000). Disadvantages for regression trees are mostly concerns about the univariate nature of splitting decisions, where an early split may lead to suboptimal later splits. Most recent procedures are built upon model predictive ability and are either based on data resampling approaches (bootstrap) or model averaging (bagging, random forests) (Leathwick et al., 2006; Cutler et al., 2007; De’Ath, 2007; Elith et al., 2008). Our approach was descriptive rather than predictive, and we therefore opted for simple tree models which are easier to interpret (De’ath and Fabricius, 2000; Olden et al., 2008). We thus followed classical procedure of growing trees and pruning via assessing complexity parameter ‘*cp*’ and chose the tree with lowest complexity within the threshold of 1 standard error of null model in order to avoid overfitting data (De’ath and Fabricius, 2000; De’Ath, 2002). We illustrated both full model tree and consequently pruned tree models. We used the rpart package from R software (R Core Team, 2012) for all regression tree analyses, see Olden et al. (2008) for a general overview of these procedures.

All the analyses produced trees that described varietal dynamics stratification to the exception of dynamics of species other than *D. alata* in the short term. In this specific analysis, none of the covariates explained variation in short term species diversity dynamics. The resulting best tree model had indeed errors greater than lack of model, independently of tree size, indicating that it was doing significantly worse at delineating covariates impacting the dependent than raw data, and had therefore no explanatory power even as an exploratory analysis. We thus dropped this model from the study results.

### Impact of Anthracnose Disease on Varietal Dynamics

To investigate the interaction between anthracnose epidemics since 1970s and its impact on varietal dynamics, we conducted regression tree analysis with number of epidemics as the dependent factor. We used our previous farm covariates and included our estimates of yam agro-diversity (number of past *D. alata* varieties cultivated, number of current *D. alata* cultivated, number of past species other than *D. alata*, number of current species other than *D. alata*) in the model in order to check their relevance in disease/agro-diversity dynamics. We then followed the above mentioned regression tree procedures, with the model describing the best covariates that segregated experience of anthracnose by the producers. We chose to keep framing the question as potential interactions between epidemics and agro-diversity dynamics and did not interpret the resulting model as direct causative links.

## Results

### Varietal Diversity: Past and Current State for Yams in Guadeloupe

**FIGURE 2:**
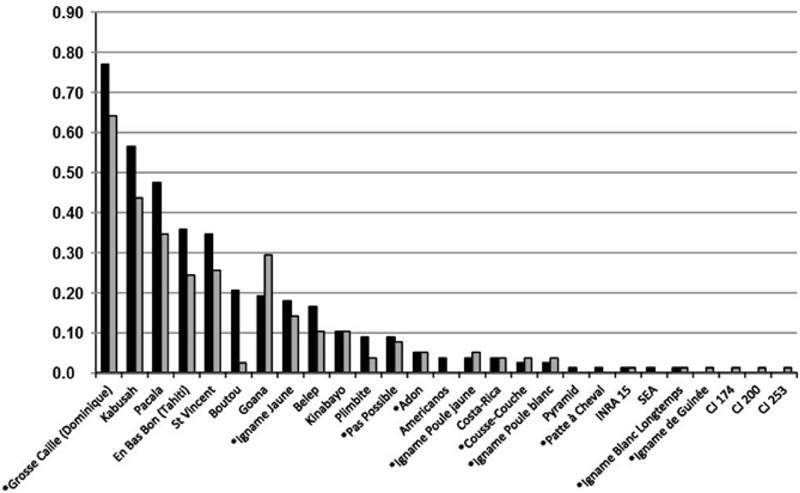
**Past and current frequencies of Guadeloupean yam varieties in study sample of producers.** Varieties are ranked by past cultivation frequency, from common to rare. Varieties with names beginning with an asterisk belong to yam species other than *D.ioscorea alata*. Varieties with coded names are most probably escapees from institutional breeding programs. In black, varieties cultivated in the past; in gray, varieties cultivated by producers in 2014.

Our sample of 78 yam producer reported an average of 1.85 (±1.48) current varieties for *D. alata* (range 0–6) compared to 2.55(±1.70) in the past (range 0–8). The total number of species other than *D. alata* currently cultivated was 1.15 (± 1.22) compared to 1.27 (±1.03) in the past. The number of *D. alata* varieties cultivated in the past was significantly greater than varieties currently cultivated (Welch *t*-test: *t* = –2.76, df = 151.00, *P* = 0.006) while there was no significant difference for species other than *D. alata* (Welch *t*-test: *t* = –0.64, df = 149.78, *P* = 0.52). Despite this decline for the majority species *D. alata*, our results demonstrated that most varieties remained in cultivation over time and varietal and species diversity were generally conserved (**Figure 2**). Some varieties had important variations in cultivation frequency, while other did not. The varietal pool under study exhibited the following trends: popular varieties from the past had the greatest rate of decline, and the observed decline attenuated for varieties less cultivated in the past. Two varieties strongly departed from this trend: “Boutou” had a strong decline, and “Goana” had a strong growth compared to past cultivation frequency. The observed trend was supported by comparing with variety census datasets from 2005 and 2009, with the exception of a few cases all belonged to species other than *D. alata* (namely “Pas Possible”, “Adon”, “Igname Poule Jaune”, “Igname Poule Blanc”, “Cousse-Couche”) mostly from the long tail of rank distribution. These species were subjected to either sampling bias variation or greater yearly variance in cultivation due to casual diversification opportunities for producers (data not shown, marginal effect). We also observed that some of the cultivated varieties declared by producers had alphanumeric names, and were actually escapees from local breeding programs before they eventually become official varieties: this practice was encouraged in the past, following a spirit of co-breeding practice, and Guadeloupean yam producers are generally prone to trying new varieties.

### Varietal Dynamics in Guadeloupe

For each question, we describe covariates order from most impacting data structuration to least impacting, describing covariates only present in unpruned tree in *italics* (these covariates are less efficient in explaining sample variance but might be interesting to discuss in regard of agro-diversity dynamics process).

#### *Dioscorea alata* Cultivated in the Past

**FIGURE 3:**
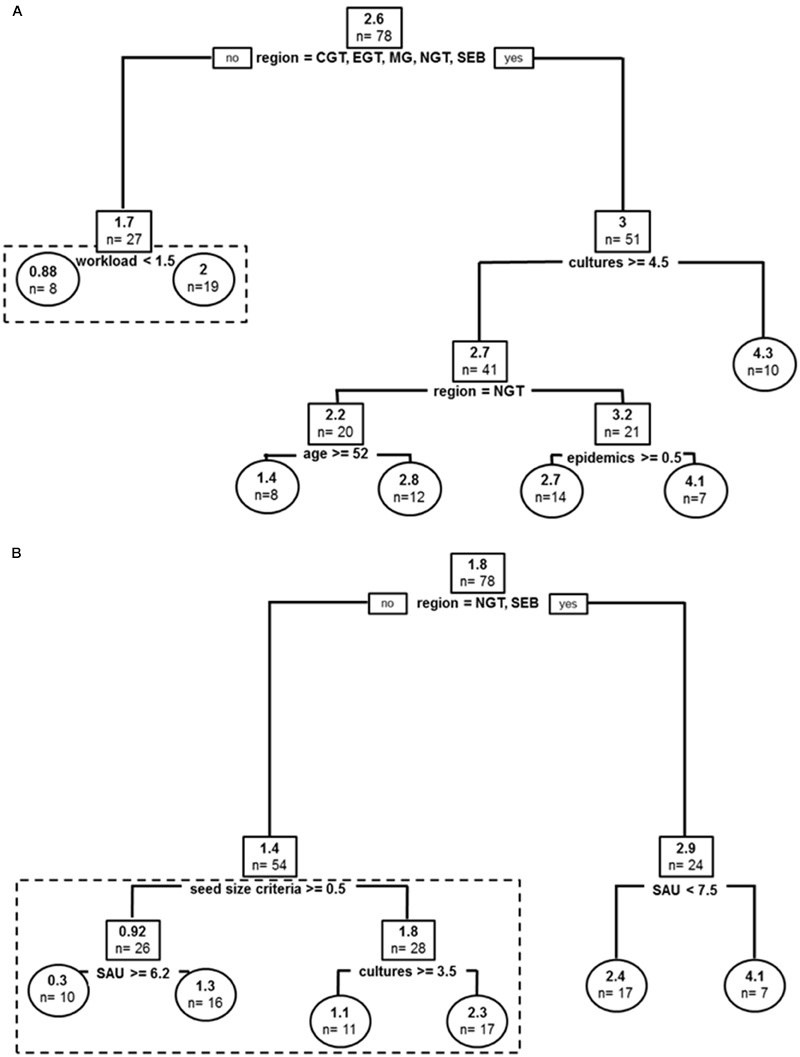
**Past and current stratifications for *D. alata* varieties cultivated in Guadeloupe: (A)** Regression tree model for *D. alata* yam varieties cultivated in the past. **(B)** Regression tree model for *D. alata* current yam varieties cultivated. Root and node labels in squares, leaf labels in circles, both indicating average number of varieties in group and sample size below. Split covariates are indicated in bold, with split condition met rightwise. Depth of branches is proportional to covariate weight (deviance). Pruned tree excludes the dotted box.

Covariates in the resulting tree were sub-divided by geographic sub-region (affecting best tree two times), cultures (i.e., number of crops grown at farm level), producer’s age, reported anthracnose epidemics and *workload* (producer strategy to devote time to yam crop) (**Figure 3A**). The global tree explained 40% of sample variance in past varietal diversity and pruned tree 37%. Producers with the highest yam agro-diversity were in peripheral areas in Guadeloupe (CGT, EGT, MG, NGT, SEB) and also had the higher on-farm diversity (number of cultures > 4.5). For producers from these sub-regions with less crops, number of epidemics segregated producers with high variety number to those with lower agro-diversity in North of Grande Terre (4.1 vs. 2.8), while age delineated producers otherwise with –older farmers cultivating two times more varieties on average. In the more central sub-regions (EBT, NBT, SGT), average number of yam varieties was lower and *workload* (i.e., ‘time alloted to yam crop’) segregated average yam agro-diversity, with producers with lower crop commitment nurturing a higher number of varieties on average (2.0 vs. 0.88).

#### Current Diversity of Cultivated *D. alata* Varieties

Covariates in the resulting tree were sub-region, surface cultivated at farms (SAU, impacting structuration two times in the resulting tree), *seed size criteria*, and *cultures* (**Figure 3B**). The global tree explains 48% of sample variance in current varietal diversity and pruned tree 22%. The two most extreme peripheral areas of Guadeloupe (NGT, SEB) hosted higher number of *alata* varieties. In these places, only farm cultivated surface (SAU) segregated agro-diversity, with farms smaller than 7.5 ha demonstrating more diversity on average. In all other locations, producers with *size criteria* for their tuber seeds had higher number of *alata* varieties: 1.8 vs. 0.92 for those who had no size criteria for tuber seeds. For producers without size criteria for seeds, SAU delineated higher number of varieties, this time with bigger farms hosting more agro-diversity. Producers with *seed size criteria* segregated in varietal diversity by number of crops, with farms with more crops also demonstrating higher number of *D. alata* varieties (1.1 vs. 2.3). Also, the average number of *alata* varieties currently cultivated is lower than in the past.

#### Species Other Than *D. alata* Cultivated in the Past

**FIGURE 4:**
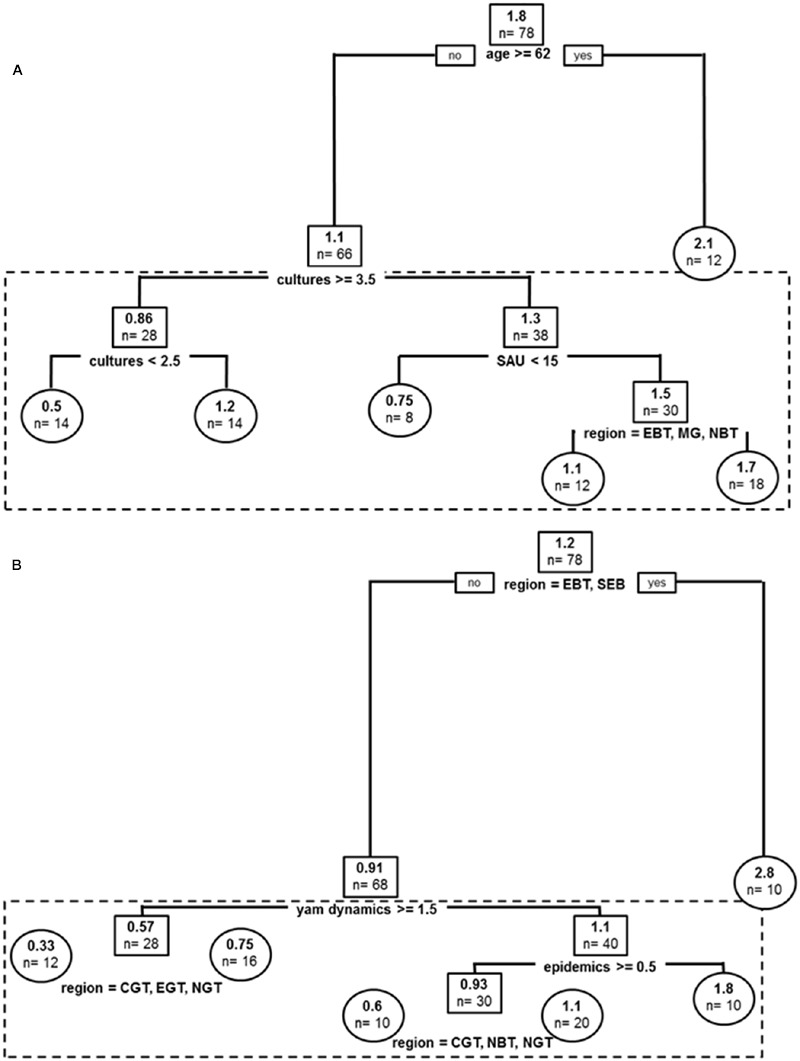
**Past and current stratifications for species other than *D. alata* cultivated in Guadeloupe: (A)** Regression tree model for yam species other than *D. alata* cultivated in the past. **(B)** Regression tree model for yam species other than *D. alata* currently cultivated. Root and node labels in squares, leaf labels in circles, both indicating average number of varieties in group and sample size below. Split covariates are indicated in bold, with split condition met rightwise. Depth of branches is proportional to covariate weight (deviance). Pruned tree excludes the dotted box.

Covariates in the resulting tree were Age, *cultures* (i.e., total number of crop species, affecting best tree two times), *farm cultivated surface* (*SAU*) and *sub-region* (without any trend in pattern) (**Figure 4A**). The global tree explains 28% of sample variance in past cultivated species and pruned tree 12%. Older producers (>62 yo) cultivated more of the other species on average (*n* = 2.1). Other producers cultivated more species on more diversified farms but the less diversified producers were also specializing on yam species other than *D. alata*. They cultivated *n* = 1.2 species on average compared to *n* = 0.5 for intermediate diversity producers. Similarly to *D. alata* yams, smaller farms cultivated higher diversity of other yam species (SAU < 15 ha), and this effect was stronger in a contracted central production basin (EBT, MG, NBT), i.e., in sub-regions of usually higher agro-diversity for yams.

#### Current Diversity of Species Other Than *D. alata*

Covariates in the resulting tree were sub-region (affecting best tree three times), *yam dynamics*, and *anthracnose epidemics* (**Figure 4B**). The global tree explains 40% of sample variance in current cultivated species and pruned tree 28%. The first sub-region split grouped EBT and SEBT, two locations from Basse Terre with climatic conditions fitting best for yam culture, especially for species other than *D. alata*, which had indeed the highest number of other species (*n* = 2.8 on average). Other producers segregated into farmers with current negative appreciation of yam crops (*yam dynamics* < 1.5) which cultivated *n* = 0.57 other yam species on average (a condition mitigated within *sub-regions*, with weaker effects in upper North, Center and East of Grande Terre –NGT, CGT, EGT). For producers with otherwise better appreciations for yams, anthracnose *epidemics* (mostly affecting *D. alata*) was impacting the number of other yams cultivated and these producers were growing *n* = 1.8 other species on average compared to producers who did not experience anthracnose. In the latter category, mitigation among sub-regions also occurred in favor of Northward locations (*n* = 0.6 vs. *n* = 1.1).

#### Varietal Dynamics of *D. alata* in the Short Term

**FIGURE 5:**
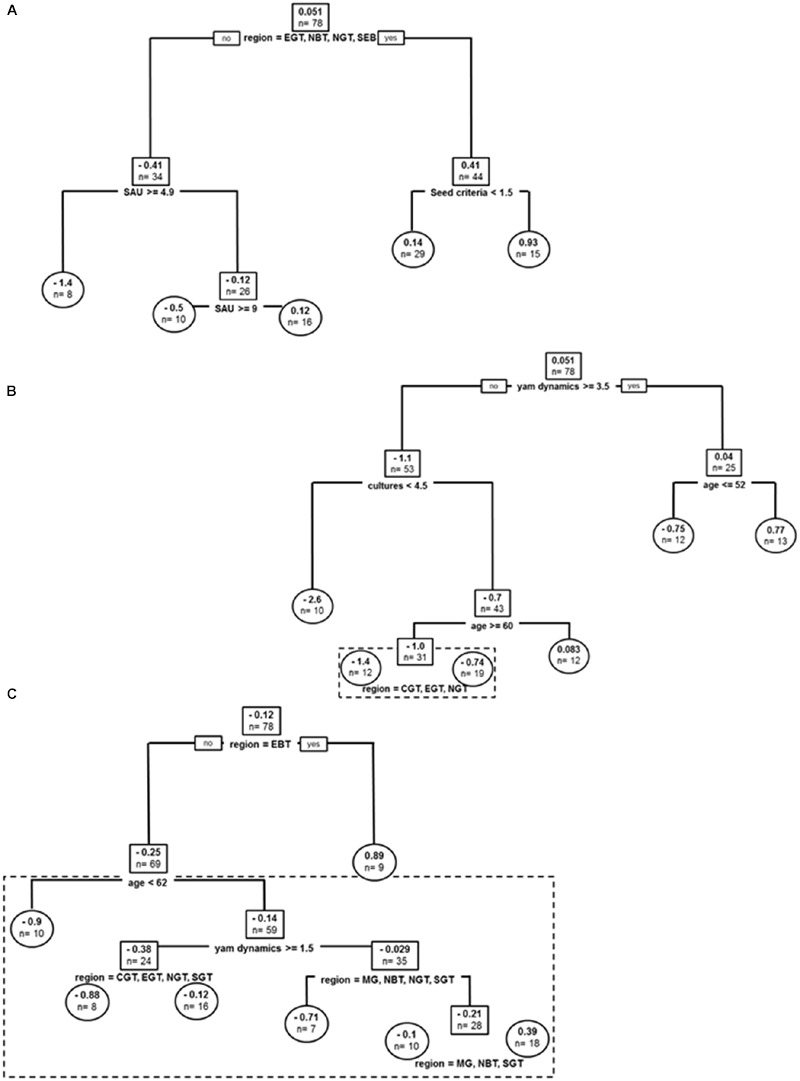
**Varietal dynamics for yams: (A)** Regression tree model for *D. alata* varietal short term (yearly) dynamics. **(B)** Regression tree model for *D. alata* varietal long term dynamics. **(C)** Regression tree model for yam species other than *D. alata* long term dynamics. Root and node labels in squares, leaf labels in circles, both indicating average number of varieties in group and sample size. Split covariates are indicated in bold, with split condition met rightwise. Depth of branches is proportional to covariate weight (deviance). Pruned tree excludes the dotted box.

Covariates in the resulting tree were sub-region (without any trend in pattern), seed criteria (segregating producers without quality or shape criteria for seeds from those who care about aspect in seeds: seed criteria < 1.5), and farm cultivated surface (SAU, impacting structuration two times) (**Figure 5A**). The tree explained 28% of sample variance in short term dynamics. The tree conveyed the idea that in some locations (CGT, EBT, MG, SGT) being less demanding about seed material helped producer regain lost varieties more quickly and thus improved the short term increase in varietal diversity, while in the other regions larger farms had better access to new varieties, probably because of an increased willingness to buy tuber seeds.

#### Varietal Dynamics of *D. alata* in the Long Term

Covariates in the resulting tree were yam dynamics (reported aims of increasing or reducing yam surface in the next years), producer’s age (impacting structuration two times in the resulting tree), and *sub-regional* impact segregating upper North, Center and East of Grande Terre (NGT, CGT, EGT; **Figure 5B**). The global tree explains 43% of sample variance in long term dynamics and pruned tree 41%. Reported yam dynamics first segregated long term loss of varieties in producers, with only the most enthusiast producers (score > 3.5) grouped in the resulting tree, while producers without goals to increase their cultivated yam surface and those declaring willingness to reduce it grouped together. Enthusiastic yam producers did not demonstrate long term loss in variety numbers on average, while the others had a net decline of 1.1 varieties. Enthusiasts pattern of diversity was actually impacted by age, with younger producers demonstrating gains of new varieties, while older producers tending to lose *D. alata* varieties. Non-enthusiast producers were structuring first depending on number of crops on farm (*cultures*), with more diversified producers being highly impacted by varietal loss in the long term (–2.6 varieties on average). Age was then the most stratifying factor, this times acting as a buffer against loss, since older producers in non-enthusiast group had a stable yam diversity, while younger producers (age < 52 yo) lost 1.0 variety on average in the long term. Finally, the latter loss dynamics was approximately two times weaker in North, East, and Centre Basse Terre (NBT, EBT, CBT), compared to the rest of Guadeloupe.

#### Diversity Dynamics of Species Other Than *D. alata* in the Long Term

Covariates in the resulting tree were sub-region (affecting best tree four times), producer’s *age, yam dynamics* and *sub-regional* impact (impacting structuration three times in the resulting tree) (**Figure 5C**). The global tree explains 27% of sample variance in long term dynamics and pruned tree 14%. East of Basse-Terre demonstrating an average gain of *n* = 0.89 in species other than *D. alata* cultivated in the long term. Older producers (>62 yo) demonstrated greater loss of species other than *D. alata* compared to younger producers (loss of *n* = 0.9 vs. *n* = 0.14). Among younger producers, commitment to yam crop was most structuring long term dynamics of these species, with a group willing to abandon the crop (*yam dynamics* < 1.5) losing an average *n* = 0.38 species (a condition mitigated within sub-regions with Grande Terre locations demonstrating a weaker impact of producers age). Above yam dynamics threshold, other species cultivated were stable, though sub-regional dynamics demonstrating diverse impact of commitment to yam with the notable exception of MG, NBT and SGT where there was overall an average increase in other species cultivation (*n* = 0.39).

### Impact of Anthracnose Disease on Varietal Dynamics

**FIGURE 6:**
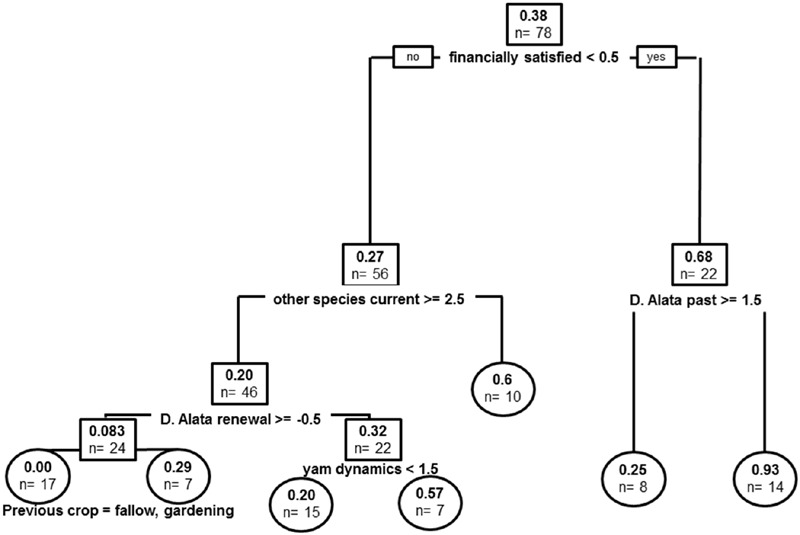
**Regression tree model for experience of anthracnose epidemics.** Root and node labels in squares, leaf labels in circles, both indicating average number of epidemics in group and sample size. Split covariates are indicated in bold, with split condition met rightwise. Depth of branches is proportional to covariate weight (deviance).

Covariates in the resulting tree were financial satisfaction, current species other than *D. alata* cultivated, past varieties of *D. alata*, renewal of *D. alata* varieties, yam dynamics and previous crop cultivated (previous plot use) (**Figure 6**). The global tree explains 36% of sample variance in anthracnose experienced levels. Experience of epidemics was more important in producers with financial dissatisfaction (*n* = 0.68 vs. *n* = 0.27 epidemics on average). These producers had generally experienced more disease when they had higher *D. alata* varietal diversity. Producers reporting financial satisfaction were first structured by number of species other than *D. alata*, suggesting a shift from the disease susceptible species toward those more tolerant of the pathogen. Further stratification in producers that did not shift toward cultivation of other species was then impacted by strategy of variety renewal, with producers that did not counter varietal loss experiencing less anthracnose epidemics than those that tried to compensate variety losses (*n* = 0.083 vs. *n* = 0.32). Producers with the first strategy were more impacted by disease when cultivating yams after fallow or market vegetable-gardening, while the latter were segregating by negative yam dynamics, suggesting that epidemics also correlated with decision to decrease commitment to yam cultivation.

## Discussion

Our results showed a decline in yam varietal diversity in Guadeloupe over the three latest decades, mostly affecting the most cultivated local species *D. alata*. Yam agro-diversity was nevertheless buffered at the regional level, with most of past varieties still in cultivation and decline affecting common varieties the most. Our analyses of variety and species dynamics revealed several structuring factors independently of species (e.g. sub-region, farm size, number of cultures at farm level and producers strategies: age, commitment to yam crop, yam dynamics). Some of these covariates seem to validate the common perception of agro-diversity as a productivity buffer strategy. Last, anthracnose epidemics affected variety dynamics and it was strongly associated with financial dissatisfaction. It had some stratified impacts on yam producers and did not affect producers equally, since some transitioned into cultivating less sensitive species. We discuss these results and highlight consequences for agricultural diversity in yams in the future.

Did yam agro-diversity decline locally over the three most recent decades? Yam agro-diversity in Guadeloupe demonstrated a decline in varietal diversity, mostly impacting the main cultivated species (*D. alata*), with producers relying on lower varietal divesity in their current cropping systems compared to past diversity (*n* = 1.85 ± 1.48 varieties vs. *n* = 2.55 ± 1.70 in the past). Other species of yam did not follow the same trend, and while less cultivated than *D. alata*, demonstrated relative stability in occurrence. These observations are consistent with the local pattern of decreased cultivation of yam crop in the latest decades. This trend nevertheless contrasted with worldwide pattern of agro-diversity loss in that decline affected generally the commonest varieties and species and infrequent varieties were still cultivated and conserved at the farm level, confirming attachment to older varieties despite productivity fluctuation and despite many of them being especially susceptible to anthracnose disease. Indeed, many producers declared they were maintaining landraces by trying to protect them and have them grow within resistant cultivar patches. The local context was thus generally conservation friendly, possibly because the yam sector is not organized and yam cropping mostly survives by producer self-agency. Patterns of diversity dynamics were nevertheless explained by sub-regional and farm characteristics and attention should focus on better understanding local risks and thus devise more efficient conservation strategies on farm. In summary, the trend in agro-diversity dynamics in our study contrasted with the observed worldwide pattern toward landraces’ loss and suggested that turn-over may be compensated by perceived tuber characteristics at the advantage of older varieties.

What factors structured past and current agro-diversity, and how did they affect loss and gain dynamics? We’ll first take a general overview of this issue, before discussing in more details two important aspects –varietal diversity as a minimal productivity insurance and short term vs. long term varietal dynamics.

Analyses of agro-diversity and diversity dynamics for both *D. alata* varieties and species other than *D. alata* emphasized covariates such as sub-region, age of producer, number of crop, total surface cultivated, yam anthracnose epidemics, yam dynamics, seed size criteria for yam as main factors (covariates used in pruned tree models, **Figures 3A** and **4B**). All of these covariates sometimes affected data structure as secondary factors in unpruned tree models, in addition to two other covariates only used in unpruned tree models: yam crop workload (**Figure 3A**) and seed size criteria (**Figure 4A**). These are mostly farm level factors (sub-region, number of crops, total surface cultivated) and producer characteristics (age, yam dynamics) and only two were relevant to cropping practice or cropping experience, namely seed size criteria and epidemics. Most agronomic covariates did not impact diversity and its dynamics: neither number of yam plots, staking, weeding, use of chemicals, frequency of chemical use, seed selection criteria, seed treatment before planting, use of fertilizer, nor economic factors or personal preferences such as readiness to invest financially in yam crop, financial satisfaction with yam crop, personal satisfaction with yam crop. These results demonstrate further risk factors for yam diversity, since producers are certainly demographically older, thus putting varieties at higher risk of loss in the future. This is reinforced by the fact that many yam producers experience yield trouble with the crop and expressed willingness to decrease cultivation (‘yam dynamics’ covariate). Sub-regional patterns have been consistent with either local climatic conditions (e.g., suiting best growth of other species in Basse Terre) or the potential distribution of economic roads toward marketplaces (central basin) but also proved asymmetries in diversity dynamics such as buffering varietal loss effects in Grande Terre (**Figure 5A**). Impact of sub-region as a covariate possibly relates to social networks of producers. Understanding the impact of interactions and seed exchanges between producers would be an interesting avenue of research since it is already known to impact agro-diversity (e.g., Alvarez et al., 2005). Indeed, while understanding producers’ reasons to preserve their agro-diversity, it is also important to focus on processes involved in emergence and dynamics of varietal diversity (Brookfield and Stocking, 1999). Our results thus suggested that varietal dynamics may be more stratified by producers’ strategies and constraints than by cropping systems or agronomics *per se*, and that producers’ networks do certainly impact variety fates.

Agro-diversity, and especially higher varietal diversity on farm, might be driven by a productivity insurance strategy. This idea was proposed several times in the literature (Smithson and Lenne, 1996; Di Falco and Perrings, 2003; Di Falco and Perrings, 2005), and might actually be mitigated by other non-exclusive hypotheses such as diversity in varieties being also associated to diversity in uses, or a producer strategy in harvest management, for e.g., early or late varieties leading to delays in crop maturity and more favorable harvest labor. Several of our results hint at the hypothesis that varietal diversity may buffer productivity factors. First, varietal diversity segregated several times with number of crops at farm level, and may be a posteriori explained as producers with otherwise high commitment to other cultures relying on stable yam harvest via greater varietal diversity (**Figures 3A,B** and **4A**). This hypothesis is reinforced by the fact that producers in such a situation have a tendency to buffer losses or renew the varieties they lost at higher rates than producers with greater specialization on cropping yams (**Figure 5B**). Along the same lines of interpretation, workload segregates past *D. alata* varietal diversity, and producers with lower commitment to yam crop had greater past diversity, so that higher diversity stratification is certainly explained by lower specific commitment to yam crop because of greater time devoted to other crops. We conclude that harvest perspective associated with diversified agro-diversity can be of special importance in orphan crops and diversification strategies of farmers, and may still be a major driver of landraces conservation effort.

Short term and long term variety renewal were strongly contrasting and related to different dynamics and processes. Short term yam dynamics centered immediate difficulties in variety renewal (variety gains seemed easier with either larger farms with less financial constraints, or to small farm producers willing to relax seed quality criteria in order to recover lost varieties, **Figure 5A**). On the other hand, long term agro-diversity dynamics was a mix of projected commitment to growing yams, producer age, and other crop constraints (**Figure 5B**). In fact, it was most impacted by producers willingness to commit to yams with only younger producers aiming to increase yam cultivation and in doing so enrich their variety pool, or older producers (indicating a cultural or historical attachment to the crop), or producers with other crop constraints as discussed above. These results illustrate possible constraints in access to planting tuber seed material in the short term, possibly due to the lack of an organized local seed industry for this crop, and a diversity of reasons to commit to variety renewal entrenched with yam crop perceived as a cultural legacy. In the long term, perspectives to conserve local agro-diversity dynamically on farm (e.g., Jarvis et al., 2011) are weakened by aging producer demographics and strongly rely on a future increase of farmers committing to the crop. This is especially important, given older farmers are crucial partners to conservation efforts (e.g., Alvarez et al., 2005). Currently, local agro-diversity in yams is mostly secured by static Genebank (CRB^1^), and varieties are available on request to farmers. Such differences in short term vs. long term variety renewal is indicative of the nature of economic constraints and perspectives for diversity, and greater research effort should focus on such issues, in order to allow for improved policies toward enhancement of agro-diversity conservation on farm.

Did recurrent epidemics of anthracnose disease since the 1970s impact agro-diversity dynamics? Anthracnose epidemics have impacted local yam diversity dynamics since the disease appearance in the Caribbean in the 1970s (when it became the main disease on yams). Indeed, the covariate appears as a stratifying factor in our regression trees, though only as a secondary factor (**Figures 3** and **4**), suggesting pattern of varietal dynamics are not directly shaped by disease occurrence *per se*. It was nevertheless strongly related with financial dissatisfaction (**Figure 6**), and probably translated in triggering the choice to decrease yam cultivation even in producers financially satisfied. Generally, increased reports of epidemics coincide with higher diversity of *D. alata*, the species most susceptible to anthracnose disease (**Figure 6**), but disease prevalence might have matched with the most susceptible varieties and may not have had adverse effects on every variety equally. It nevertheless impacted producers in a mixed fashion, and made some producers engage into strategical shift toward other species known to be more resistant, since producers cultivating more of these species currently also had greater rates of reported disease in the past (**Figure 6**). The disease is known to have impacted varietal strategies by producers: it became an important concern and altered disease management practices, especially in producers with high varietal diversity (Penet et al., 2016), ever since local dispersal falls within natural rain dispersal range in fields (Penet et al., 2014). In summary, while diseases are thought of as the focal trigger of variety turn-over, there are other processes at play and diseases are actually more than mere drivers of varietal obsolescence via harvest decrease.

Our study explored the dynamics of yam varietal and species diversity at farms level with a mostly experienced, middle-age to late-career sample of producers. Our sample allowed us to investigate diversity dynamics of producer choices in the most recent three decades based on agronomic options and farm context. We emphasized a difference between short term dynamics which seemed mostly based on seed quality/economic constraints, over long term varietal dynamics that was mostly impacted by agronomy, producer age and sub-regional factors. While the future of local yam diversity is rather mitigated, dependent on unpredictable turn-over of the pool of farmers and cultural/historic appreciation of varietal diversity in the next generation of producers, there’s hope with perspective on consumers’ side. Indeed, consumers’ choices and preferences have been shown to be leaning toward more local products, even at higher prices or under label constraints (e.g., organic), and a diversity of tastes only brought up by varietal and species diversity (Barlagne et al., 2015, 2016). These demands might buffer variety loss despite disease constraints, as they rely on the maintenance of a diversified offer. Finally, our results highlight the need for greater prospect of varietal dynamics in diverse crops and agricultural contexts worldwide, as knowledge of crop genetic diversity and impact of evolutionary agro-ecological forces also need to be combined with the analysis of loss and gain in varieties and how they all structure agro-resources availability and evolution.

## Author Contributions

Contributed to project definition and funding: LP, DC, JB, AA, EB, FB, SG, CP, DP. Contributed to data acquisition: LP, EB, JB. Contributed to analyses: LP. Contributed to writing and drafting: LP, DC, JMB, AA, FB, SG, CP, DP.

## Conflict of Interest Statement

The authors declare that the research was conducted in the absence of any commercial or financial relationships that could be construed as a potential conflict of interest.

## Footnotes

1 http://intertrop.antilles.inra.fr/Portail/pages/crb-ptda
